# Which outcomes should be included in a core outcome set for capturing and measuring doctor well-being? A Delphi study

**DOI:** 10.1136/bmjopen-2024-094973

**Published:** 2025-05-13

**Authors:** Gemma Simons, Naomi Klepacz, David S Baldwin

**Affiliations:** 1 University of Southampton Faculty of Medicine, Southampton, UK; 2 Hampshire and Isle of Wight NHS Foundation Trust, Southampton, UK; 3 Salisbury NHS Foundation Trust, Salisbury, UK; 4 NIHR Applied Research Collaboration Wessex, Southampton, UK; 5 School of Health Sciences, University of Southampton, Southampton, UK; 6 Department of Psychiatry and Mental Health, Department of Medicine, University of Cape Town, Observatory, Western Cape, South Africa

**Keywords:** MENTAL HEALTH, Health Workforce, Delphi Technique

## Abstract

**Objectives:**

To develop a core outcome set (COS) to capture and measure the well-being of doctors working in the National Health Service (NHS).

**Design:**

An online Delphi study.

**Setting:**

UK NHS.

**Participants:**

Participants from four stakeholder groups: (1) those who might use the COS in research, (2) organisations that measure/capture NHS staff well-being, (3) professionals with experience managing NHS staff well-being and (4) NHS doctors were identified through authorship of relevant publications, attendee lists of doctor well-being conferences and meetings, professional bodies, participation in a previous study and recommendations from others. They were recruited via email.

**Interventions:**

A two-stage process: (1) creating a list of 43 well-being outcomes informed by a systematic review of well-being measurement instruments, a survey of UK doctors and two doctor engagement workshops and (2) an online Delphi study (with two rounds) to reach consensus. Outcomes were rated on a 9-point Likert scale; ‘consensus’ was reached when ≥75% agreed that an outcome was critical for inclusion in the COS.

**Results:**

52 participants completed both Delphi rounds. Seven well-being outcomes met the threshold for inclusion in the COS: general well-being, health, personal safety, job satisfaction, morale, life work balance and good clinical practice.

**Conclusions:**

Use of the COS has the potential to reduce heterogeneity and standardise the capture and measurement of doctor well-being, and ensure outcomes important to all stakeholders are reported.

**Trial registration:**

This study was prospectively registered with the Core Outcome Measures in Effectiveness Trial initiative at www.comet-initiative.org (Registration: 1577).

**STRENGTHS AND LIMITATIONS OF THIS STUDY**

- A salutogenic and consensus approach was used to achieve agreement between four stakeholder groups.
- There was no internationally agreed definition of doctor well-being available to use for this study.
- The study protocol was developed following the Core Outcome Measures in Effectiveness Trial criteria.

## Introduction

 Doctors globally are increasingly experiencing high workloads and challenging working conditions and, consequently, are reporting high levels of stress, anxiety, depression,^1^ emotional distress, burn-out risk^2 3^ and suicidal feelings.^4 5^ This negatively impacts patient care quality, safety and satisfaction^6^,^8^ and leads to declining job satisfaction and doctors leaving the workforce.^9^ In a UK context, the 2023 National Health Service (NHS) staff survey^10^ found that 44% of doctors felt unwell in the previous 12 months because of work-related stress, and 38% often or always found their work emotionally exhausting. Emphasis is often placed on doctors to be more resilient, with stigma and a fear of potential repercussions preventing doctors from speaking up about their well-being.^11^ However, there is an emerging consensus that some aspects of doctors’ training, working conditions and organisational support negatively impact well-being.^5^ The well-being of doctors significantly impacts workforce planning, cost, healthcare quality and patient outcomes.^12^ Dissatisfaction with role/place of work or NHS culture was cited as the top reason for leaving the workforce in a General Medical Council survey,^13^ with burn-out/work-related stress as the third most cited reason behind retirement. Poor mental well-being of staff is estimated to cost the NHS at least £12.1 billion per year; tackling poor mental well-being and reducing the number of staff leaving the NHS could save up to £1 billion.^14^ The UK’s health system prioritises patient care—often over staff well-being—but long-term patient care and safety depend on staff well-being.^15^

The need to address doctor well-being is well recognised, with government and industry reports highlighting the need for improvement.^28 11 15^,^18^ While recognising the urgent need to address doctors’ well-being, these reports often fail to operationalise well-being or specify the outcome or measurement tools required to gauge the success of their recommendations. For example, the ‘NHS Long Term Plan’^19^ aims to make the NHS ‘the best place to work’ but provides little detail on implementation or how success will be captured or measured. The NHS Long Term Workforce Plan^20^ commits to implementing actions from the NHS People Plan^21^ to ensure that staff have access to well-being services and support; however, the British Medical Association has questioned how this ambition will be made a reality.^22^ Many employers and education deaneries now provide well-being programmes for doctors and implement the NHS health and well-being framework.^23^ However, evidence of the success of these (and similar) programmes—often aimed at individual coping strategies, resilience and productivity—suggests limited effect.^12^ The lack of consensus on what doctor well-being is and how it should be measured means that the monitoring and evaluation of these strategies are inconsistent.

The ongoing and accurate measurement of doctors’ well-being is necessary to understand local and specific needs and ensure the effective delivery of staff services.^17^ It is, therefore, vital for both research and governance to take a consistent data-driven and evidence-based approach to doctors’ well-being, taking account of the many dimensions (ie, social, cultural, environmental and economic) and levels (ie, individual, organisational and societal) that comprise this complex issue. However, work has not yet been undertaken to standardise the definition and measurement of doctor well-being. In addition, ‘well-being’ has often been used interchangeably with, or to describe, mental health, with previous research focusing largely on ‘pathologies’ such as depression, anxiety and burnout rather than positive measures of well-being. Consequently, workplace well-being has become a measure of the absence of mental health disorders. A salutogenic approach^24 25^ that measures positive determinants, context, mechanisms and individual and group well-being should be preferred when considering doctor well-being.^26^ A salutogenic approach is a positive approach that looks prospectively at how to create, enhance and improve well-being; as opposed to a pathogenic approach that looks retrospectively at the disease burden of doctors (usually mental health diagnoses).

Our systematic review^27^ found well-being outcomes and measurement tools used in doctor well-being research were heterogeneous, demonstrating the need for a core outcome set (COS). COSs are consensus minimum groups of outcomes with recommended reliable and valid measurement tools. Reaching agreement among stakeholders—including NHS doctors—ensures a consistent and comprehensive focus, facilitating comparison between organisations through the generation of ‘big data’, and in doing so, provides decision-makers with the evidence needed to inform future workforce strategies, interventions and actions. We used a salutogenic and consensus-based approach to develop a COS to capture and report on the well-being of doctors in the NHS. To our knowledge, this study represents the first time a non-pathological concept—well-being—has been applied to the COS-STAndards for Development (COS-STAD) guidance.^28^

## Methods

### Study overview

**Figure 1:**
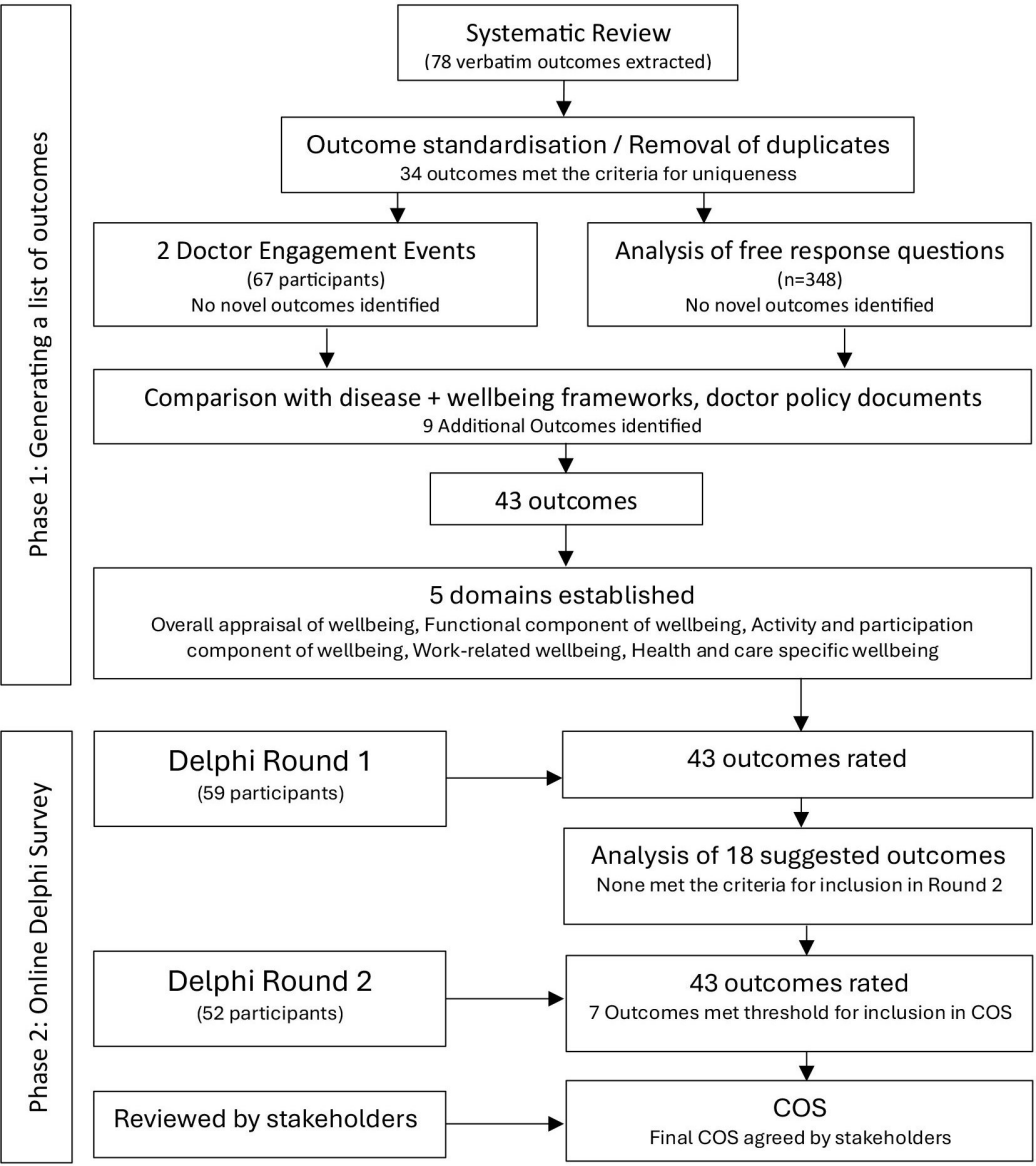
Flow diagram of the process for developing the core outcome set (COS).

The COS was developed in two stages: (1) the generation of a long list of outcomes and (2) an online Delphi survey (figure 1). The study protocol was developed following the Core Outcome Measures in Effectiveness Trial (COMET) criteria^29^ and was prospectively registered with the COMET initiative^30^ (Registration: 1577). The study is reported using the COS-STAndars for Reporting guidance.^31^

### Phase 1: generating a list of outcomes

The long list of outcomes was generated from a systematic review of doctor well-being definitions and measures,^27^ local and national doctor involvement/engagement events^32^ and analysis of free-text response questions from a national online survey of doctor well-being.^32^ Inductive constant comparison analysis^33^ allowed the participants to generate themes. As part of this ‘open coding’ technique, participants’ own words were used for code names, and the themes and meanings were constructed after the data had been collected using convergent thematic analysis.^34^ Finally, any duplicates were removed, and similar concepts were merged. Outcomes that were pathologies, symptoms of pathologies or negative in nature were removed from the long list. Uniqueness was established when the published definition of an outcome differed conceptually from those of other outcomes.^35^ Outcomes identified as unique were compared with the outcomes and domains in health, health-related quality of life, disease, and well-being frameworks,^36^,^39^ and doctor policy documents^2140^,^44^ to identify any further key concepts. Plain English descriptions—presented as ‘help text’ in the Delphi survey—were written for each outcome, guided by the published literature (table 1; online supplemental material 1). We established relevant domains by grouping thematically similar outcomes that captured a broader concept using a well-being framework developed for this study.^32^ ‘Think aloud’ interviews were conducted (n=3 doctors, n=2 psychologists) to review the uniqueness of the outcomes and the clarity, conciseness and accessibility of the ‘help-text’ definitions for stakeholders.

**Table 1:** The final core outcome set

Domain	Outcome	Description	Stakeholders rating outcomes as ‘critical’ (%)
Overall appraisal	General well-being	A state of positive feelings/affect/happiness and meeting full potential in the world (being the best person you can be in society). It can be measured subjectively and objectively using a salutogenic (positive) approach.	97.9
Functional component of well-being	Health	Subjective, or objective, evaluation of state of complete physical, mental and social well-being, not merely the absence of disease or infirmity (eg, the beneficial effects of green spaces, ability to relax).	75.0
Work-related well-being	Personal safety	Subjective, or objective, ability to go about work, and get to and from work, free from threat and safe from physical or psychological harm (infection, radiation, bullying, theft, assault).	77.1
	Job satisfaction	Subjective, or objective, evaluation of how much they like their choice of work profession, specialism, roles.	85.4
	Morale	Subjective, or objective, evaluation of feelings about the future, ability of an individual, group or organisation to have and meet shared goals/values.	83.3
	Life work balance*	Subjective, or objective, quantity, quality, and equity of time away from work and at work, the salience/clarity of the roles (the ability to work flexibly).	93.8
Health and social care-specific well-being	Good Clinical Practice	Subjective, or objective, assessment of ability to engage with high-risk cases, following standards where appropriate not rigidly, use diagnostic tests and treatments when clinically indicated and evidence based, not just in case.	89.6

* Work life balance is the preferred terminology in most literature. Placing life before work was a conscious decision by the authors for this outcome and will impact the future outcome measurement instrument selection. Further details can be found in Simons (2022).^32^

### Phase 2: online Delphi survey

The long list of outcomes was used to populate an online Delphi survey, delivered using the DelphiManager platform.^45^ The Delphi approach is a method widely used in developing COSs.^45 46^ The method aims to achieve consensus through the collection and synthesis of stakeholder opinions.

#### Stakeholder recruitment

A purposive sampling method was used to recruit participants from four stakeholder groups: (1) those who could use the COS in research, (2) representatives of organisations that measure doctor well-being in the NHS, (3) professionals with experience in managing doctor well-being and (4) doctors working in the NHS (all specialities and grades). Researchers of doctor well-being were identified through academic conference programmes and abstracts, and authorship of relevant publications. Organisations measuring doctor well-being were identified through reports and publications on doctor well-being, discussions at national group meetings (ie, Royal College of Physicians Flexibility and Well-being Group; BMA Well-being Support Stakeholder Group) and responses from a previous survey.^32^ (A full list of organisations can be found in online supplemental material 3) Professionals with experience managing doctor well-being were identified through policy documents and attendee lists from national well-being group meetings (ie, Practitioner Health Programme; BMA Support). Doctors who had participated in the doctor involvement/engagement events or our online survey and had given permission to be approached were invited to participate. Additionally, and with the permission of the group administrator, doctors who were members of the BMA Well-being Stakeholder group and Royal College of Physicians Flexibility and Well-being groups were invited to participate. We further identified stakeholders for all groups through recommendations from others. All potential participants were emailed an invitation to participate in the Delphi survey with a participant information sheet, a URL link to DelphiManager and a link to the study information video. Participants were required to provide written consent before registering their details (name and email); they did this by reading the participant information sheet on DelphiManager and checking a box to confirm consent to participate. All participants were assigned a study ID at registration, meaning data were anonymous at the point of collection.

### Delphi survey and analysis

The 43 well-being outcomes were listed with plain English descriptions by domain in a Delphi survey conducted over two rounds (round 1 took place July 2021 and round 2 in August 2021). We asked participants to rate the importance of including each outcome in the COS using a 9-point Likert Scale. Ratings were grouped into three categories: a score of 1–3 on the Likert scale indicates the outcome is of ‘limited importance to include’, a score of 4–6 indicates the outcome is ‘important, but not critical’ for inclusion in the COS, and a score of 7–9 indicates that the outcome is critical to capture and measure in the COS. Participants had the option to declare they were unable to score an item. There was a free-text comment box, and participants were encouraged to provide a rationale for their selection or additional comments. At the end of each Delphi Round, participants had the opportunity to suggest additional outcomes for possible inclusion in the COS; they were instructed that these should not be a symptom, sign or disease, nor a determinant of well-being. The criterion for considering an outcome for inclusion was that the definition of the outcome in the literature should differ conceptually from the help text offered for the existing outcomes. Where additional outcomes were suggested, the participant was emailed with the justification for inclusion or exclusion and given the opportunity to provide additional evidence/explanation.

In round 2, the percentage of participants giving each rating for an outcome was fed back to participants. Participants were also reminded of their own ratings for each outcome from round 1. Participants had the opportunity to re-rate each outcome based on this feedback. Participants were sent three reminder emails to complete rounds.

The *a priori* definition of consensus was when ≥75% of participants rated an outcome as ‘critical for inclusion’ (ratings 7–9). This definition aligns with those used in other COS development studies^47^,^50^ and the Grading of Recommendations Assessment, Development and Evaluation working group.^51^ The outcomes that met the threshold for inclusion in the COS were emailed to participants with an invitation to provide further comments on the COS.

### Patient and public involvement

Patient and public representatives, doctors and well-being experts contributed to the study’s design and helped identify potential applications of the COS.^32^ Public representatives participated in data collection and interpretation as members of organisations that capture and measure NHS staff well-being or use the COS. The COS will be disseminated through open-access events and resources that are accessible to both patients and the public.

## Results

### Phase 1: generating a list of outcomes

Our systematic review is described in detail elsewhere.^27^ A total of 78 *verbatim* well-being outcomes were extracted from the systematic review. To confirm uniqueness, these outcomes were grouped using definitions created from the research and policy literature (online supplemental material 2). Thirty-four outcomes were found to meet the criteria for uniqueness. Of these 34 outcomes, 25 outcomes are synthesised; that is, they are composed of multiple verbatim outcomes with the same conceptual definition (eg, ‘health’, ‘self-esteem’). The remaining nine could not be grouped with any other verbatim outcomes and could only be described by a single term and definition (these included ‘satisfaction with patient care’, ‘psychological safety’ and ‘compassion satisfaction’). No new outcomes were extracted from the doctor engagement/involvement events, and free-text responses from the national survey, as these verbatim outcomes were all found to be among those already extracted from the systematic review. Next, the 34 unique outcomes were compared with existing frameworks and policy documents, which resulted in the identification of a further 9 outcomes, giving a final long list of 43 unique well-being outcomes (online supplemental material 1). This long list was then examined to identify whether subgroups or themes existed between the outcomes. Five domains were identified: (1) overall appraisal of well-being, (2) functional components of well-being, (3) activity and participation components of well-being, (4) work-related well-being and (5) health and social care specific well-being.

### Phase 2: Delphi survey

Invitations to participate were sent to 72 individuals across the four stakeholder groups. Sixty participants registered for the study: giving a response rate of 83.3%. One participant withdrew, meaning 59 participated in round 1, of which 56 participants rated every outcome. Of the 59 participants, 52 also participated in round 2: giving a retention rate from round 1 to round 2 of 91.2%. In round 2, 51 participants rated every outcome. All rated outcomes were included in the analysis. Of these participants, 35% were male and 80% were doctors, of which 54% were consultants, 32% GPs, 7% associates specialists and 7% training grade doctors.

In round 1, 14 suggestions were made for additional outcomes from 8 participants (online supplemental material 4). None of these met the criteria for inclusion into round 2. Of the suggested outcomes, 18 were not novel and had already been captured by existing outcomes, and 4 were interventions, 1 suggestion ‘going on holiday’ was classified as both an intervention and not novel, being captured under ‘life work balance’ and ‘job plan/rota satisfaction’.

At the end of round 2, seven well-being outcomes met the ≥75% threshold for inclusion in the COS for capturing and measuring doctor well-being: general well-being, health, personal safety, job satisfaction, morale, life work balance and good clinical practice (table 1). The ratings for all outcomes may be seen in online supplemental material 5. We sent the agreed set of outcomes to the stakeholder participants for review. They approved the COS without amendment.

## Discussion

We have developed a COS for capturing and measuring the well-being of doctors working in the NHS. By using a salutogenic and consensus approach, we have achieved agreement among four key stakeholder groups (researchers, organisations that measure NHS doctor well-being, professionals with experience managing doctor well-being and doctors). To our knowledge, this is the first time the COS-STAD guidance^28^ has been applied to the non-pathological concept of well-being, demonstrating that it is feasible to capture and measure. Our systematic review revealed heterogeneity between well-being outcomes and measurement tools.^27^ Implementing the COS will ensure a consistent and comprehensive focus for doctor well-being data collection and prevent duplication, facilitate the generation of big data and enable comparison at organisational, local and national levels. We recommend that future research, organisational and governance measurements of NHS doctors’ well-being use this COS. This does not preclude other outcomes from being measured as required but rather represents the minimum that should be captured and measured. Further research as to how best to operationalise and measure these well-being outcomes is now needed.

The long list of outcomes presented to stakeholders was evidence-based, drawing on doctor-specific research and the broader well-being literature.^27^ The finding that the listed outcomes already represented the additional well-being outcomes suggested by participants in Delphi round 1 suggests that the list was inclusive and holistic. While several of the outcomes from the COS align with the existing NHS Staff Survey themes^52^ and questions, suggesting the COS is relevant and acceptable, other outcomes are not well represented. For example, the outcome personal safety is captured through question 13 (experiencing physical violence at work) and Q14 (harassment, bullying or abuse at work), which aligns with the People Promise^53^ theme ‘*We are safe and healthy’*. Similarly, morale is captured through questions relating to work pressure, stressors and intention to leave, which may also be viewed as negative indicators of the outcome job satisfaction, which in the NHS Staff Survey is measured through motivation (Q2) under the ‘*staff engagement*’ theme. Work life balance (Q6b–d) has been included in the NHS Staff Survey since 2021,^54^ under the theme ‘*We work flexibly*’. General well-being is captured as emotional exhaustion and burnout, taking a pathogenic rather than salutogenic approach to well-being. The outcome health is also poorly represented, with a single mental health item (Q11c) and physical health item (Q11b). While the survey does not explicitly measure good clinical practice, it captures potentially related elements such as patient safety and quality, workplace culture, ethical practice, and managerial support and training. The COS highlights where adjustments may be needed in existing data sources to ensure all key outcomes are adequately captured and measured.

It was noted that outcomes from the ‘activity and participation’ domain did not meet the threshold for inclusion in the COS. Outcomes under this domain include ‘positive relationships’, ‘recreational activity’, ‘diet’ and ‘physical activity’, also ‘engaging with preventative medicine’, including vaccination and medical screening. These outcomes require input and action from the individual, an understanding of the risk and severity of the threat to their well-being, and awareness of the benefits of acting.^55^ Outcomes included in the COS are arguably determined by the context or system in which an individual works. However, activity and participation outcomes were commonly studied in the literature as primary and secondary interventions, for example, mindfulness training and practices^56^,^60^ and debriefing^61^ and dialogue groups.^62 63^ Further consideration should be given to this domain in relation to doctor well-being.

There are a number of strengths to our study. To our knowledge, we developed the first COS for capturing and measuring doctor well-being and did so using the robust methodology set down in the COS-STAD guidelines.^28^ The COS is the product of consensus opinion from four stakeholder groups, including NHS doctors, but it is the involvement of representatives from professional bodies (ie, British Medical Association, General Medical Council, Royal College of Physicians) that is a particular strength of this study. The uptake and use of a COS have traditionally been poor, and further research is needed to understand how uptake might be improved more broadly.^64^ By including representatives from professional bodies, outcomes relevant to this group were considered in the creation of this COS. However, we acknowledge that some groups—for example, resident doctors—were under-represented and that stakeholders outside the present panel might have differing views from those of the final consensus. The number of participants in this study was acceptable for a Delphi study,^29^ and the >90% retention rate means attrition bias was not present. We ensured that the domains were not predetermined but led by the list of outcomes and their descriptions. However, the lack of an agreed definition of doctor well-being is a limitation. The published literature guided each outcome’s description, and we found variation in how outcomes were described in the literature, with some being described by a single term and others by multiple terms. For example, the outcome ‘well-being’ had the most (n=19) non-unique terms extracted from the literature (ie, general well-being, personal well-being, mental well-being, physical well-being, professional well-being, physician well-being, etc). Without an international consensus operational definition of doctor well-being, classification of outcomes and domains is subjective. To mitigate this, we published an operational definition of wellbeing^26^ derived from our literature review.^27^ Some outcomes posed a particular challenge as they did not fit the definition of well-being used for this study.^26^ Resilience is generally considered a ‘toxic term’ in medical culture.^65 66^ However, it was included for completeness as it features so heavily in policy and research literature on doctor well-being.

This study aimed to create a COS for doctors working in the NHS, and accordingly, stakeholders were UK-based. While this COS might have some international relevance, it will be important to evaluate the acceptability and applicability of this COS for doctors in other countries, particularly where healthcare systems differ from the UK. The outcomes to emerge from the Delphi study are not necessarily unique to doctors, as research suggests that causes and interventions for poor mental well-being are not necessarily profession-specific.^67^ The robust methodology we have applied in this study could be repeated to establish the degree to which this COS could be used to capture and measure the well-being of other healthcare professionals. Our next step is to identify and agree on measurement tools and criteria. This would further enhance the quality and consistency of data collected using this COS.

## Conclusions

This COS is a stakeholder-derived set of outcomes that are most important to capture and measure for doctors’ well-being. Its application in research and health service governance will reduce heterogeneity and allow for better synthesis of evidence underpinning future workforce well-being policies and interventions, potentially improving doctor well-being and workforce retention. Research is needed to identify and evaluate outcome measurement instruments.

## Supplementary material

10.1136/bmjopen-2024-094973 online supplemental file 1

10.1136/bmjopen-2024-094973 online supplemental file 2

10.1136/bmjopen-2024-094973 online supplemental file 3

10.1136/bmjopen-2024-094973 online supplemental file 4

10.1136/bmjopen-2024-094973 online supplemental file 5

## Data Availability

All data relevant to the study are included in the article or uploaded as supplementary information.
