# Supplementary material for: Which outcomes should be included in a core outcome set for capturing and measuring doctor well-being? A Delphi study
Source: BMJ Open. 2025 May 13;15(5):e094973. doi: 10.1136/bmjopen-2024-094973 (PMC12083382; doi:10.1136/bmjopen-2024-094973)
Supplement: online supplemental file 1 [file bmjopen-15-5-s001.docx]

**Supplementary Materials 1**

| **Table 1. Outcomes and their descriptions by domain** | | |
| --- | --- | --- |
| **Domain** | **Outcome** | **Description** |
| Overall appraisal of wellbeing | General Wellbeing | A state of positive feelings/affect/happiness and meeting full potential in the world (being the best person you can be in society). It can be measured subjectively and objectively using a salutogenic (positive) approach. |
|  | Meaning in life | Separate concept to wellbeing, subjective sense of purpose, engagement with a philosophy of life, or life-goals, and fulfilment. |
|  | Life satisfaction | Separate concept to wellbeing, subjective appraisal of how much the person likes the life they lead; one of the indicators of quality of life. |
|  | Quality of life | Separate concept to wellbeing, subjective appraisal of individuals position in life in the context of the culture and value systems in which they live and in relation to their goals, expectations, standards, and concerns. |
|  | Wellness | Separate concept to wellbeing, subjective or objective evaluation of the active pursuit of behaviours, choices and lifestyles that lead to a state of holistic health. |
| Functional component of wellbeing | Vitality | Relaxed possession of energy (physical, mental, and emotional) and vigour; it is not actively strived for. |
|  | Optimism | Hopeful transcendence beyond (rising above) immediate circumstances. |
|  | Personality | Observable enduring characteristics/dispositions/tendencies to engage in certain patterns of behaviour. |
|  | Health | Subjective, or objective, evaluation of state of complete physical, mental, and social wellbeing, not merely the absence of disease or infirmity (the beneficial effects of green spaces, ability to relax, for example). |
|  | Physiological function | Objective (snapshot) of body functions i.e., Electroencephalography (EEG), Heart Rate Variability, Electro-dermal activity (temperature, sweating, cortisol levels). |
|  | Cognitive function | Objective evaluation of domains such as, but not limited to, Attention, Memory, and Processing speed. |
|  | Self-esteem | Self-acceptance, self-worth (pride), sense of coherence (ability to predict events, belief in ability to manage them, that it is worth the effort, ability to be their true self, confidence in other achievements in non-work-related activities). |
|  | Sleep | Subjective, or objective, evaluation of duration, quality, and sense of feeling restored. |
|  | Financial security | Objective ability to pay for satisfactory accommodation, bills, care of dependents, ability to save for retirement, ability to cope with a sudden fall in income, ability to pay unexpected but necessary expenses. |
| Activity and participation component of wellbeing | Novelty | Subjective, or objective, growth through new experiences, learning (including post traumatic growth). |
|  | Positive relationships | Subjective, or objective, assessment of beneficial human connections (family and friends). |
|  | Sexual wellbeing | Subjective, or objective, assessment of sense of self and body, appreciating feelings of pleasure and desire, developing, and maintaining mutually respectful gender equal relationships, safe and pleasurable sexual interactions. |
|  | Recreational activity | Subjective, or objective, evaluation of the ability to participate and participation in non-work/leisure activities and the qualities of those chosen activities (example determinants are local investment and environment). |
|  | Diet | Subjective, or objective, evaluation of the nutritional content, quantity, and timing. |
|  | Physical activity | Subjective, or objective, assessment of the ability to participate in physical activity and the quality and quantity of physical exercise. |
|  | Engagement with preventative medicine | Subjective, or objective, assessment of participation in screening programmes they are eligible for and vaccines, accessing timely treatment. |
| Work-related wellbeing | Financial reward satisfaction | Subjective, or objective, evaluation of ability to receive gratification from financial reward for effort (for example satisfaction with pay and pension). |
|  | Personal safety | Subjective, or objective, ability to go about work, and get to and from work, free from threat and safe from physical or psychological harm (infection, radiation, bullying, theft, assault). |
|  | Psychological need satisfaction | Subjective, or objective, assessment of how autonomy (being in control of your life, work) belonging and competence needs have been supported by colleagues (inclusive, positive culture), managers (adequate workforce allow development), supporting services (IT, administration, legal, occupational health). |
|  | Psychological safety | Subjective, or objective, evaluation of the consequences of taking interpersonal risk at work (trust, information sharing). |
|  | Job satisfaction | Subjective, or objective, evaluation of how much they like their choice of work profession, specialism, roles. |
|  | Morale | Subjective, or objective, evaluation of feelings about the future, ability of an individual, group or organisation to have and meet shared goals/values. |
|  | Engagement | Subjective, or objective, assessment of involvement and absorption with, commitment to, work. |
|  | Life work balance^[[1]](#footnote-1)^ | Subjective, or objective, quantity, quality, and equality of time away from work and at work, the salience/clarity of the roles (the ability to work flexibly). |
|  | Workability | Timely, objective assessment of having occupational competence and virtues, the health required for competence in an appropriate work environment by appropriate occupational health professionals. |
|  | Self-care | Subjective, or objective, assessment of behaviours to look after own health and wellbeing at work (taking breaks, time off work for sickness), accessing appropriate support services, adequate resources (estates, workforce, rapid-access self-referral services) to support this. |
|  | Professional Development | Subjective, or objective, assessment of ability to participate and engage with learning and teaching knowledge and skills, and to progress. |
|  | Identification with work | Subjective, or objective, assessment of value and meaning assumed by the individual, or a group/team, at work (pride in work, professional identity). |
|  | Resilience | Subjective, or objective, individual, or group level, preservation of, or return to, previous function after exposure to trauma. |
|  | Emotional intelligence | Subjective, or objective, self-awareness, self-management, social awareness, and relationship management. |
|  | Voice and influence | Subjective, or objective, assessment of ideas, concerns and expectations expressed informing policy and practice. |
|  | Confidence in leadership | Subjective or objective assessment of government and management competence, transparency and compassion, inclusivity, engagement and empowerment of those they are responsible and accountable for. |
|  | Recognition satisfaction | Subjective, or objective, evaluation of appreciation by colleagues, patients, public, government (civility). |
| Health and social care specific wellbeing | Compassion satisfaction | Subjective evaluation of ability to receive gratification from caregiving to patients, patients' families, colleagues (satisfaction with non-financial rewards of the work). |
|  | Altruism | Subjective, or objective, evaluation of selfless concern for the wellbeing of others (patients and colleagues). |
|  | Satisfaction with patient care | Subjective, or objective, assessment of quality of health and social care their patients receive from themselves and others (impacted by things such as staffing levels, competence, equipment, estates and funding available). |
|  | Job plan/rota/rotation satisfaction | Subjective, or objective, evaluation of ability of role, responsibilities/rota/breaks to account for the quantity, types, of work (workload), the intensity, duration, of physical, mental, and emotional demands and the rest/activities/resources needed to maintain it. |
|  | Good clinical practice | Subjective or objective assessment of ability to engage with complex or challenging patients/cases and advocate for them as indicated and in an evidence-based way. |

1. Work life balance is the preferred terminology in most literature. Placing life before work was a conscious decision by the authors for this outcome and will impact the future outcome measurement instrument selection. Further discussion can be found in 32. Simons G. How should wellbeing be measured in UK doctors? A salutogenic, consensus approach, towards a Core Outcome Set for doctor wellbeing measurement. University of Southampton, 2022. [↑](#footnote-ref-1)
