# Supplementary material for: Which outcomes should be included in a core outcome set for capturing and measuring doctor well-being? A Delphi study
Source: BMJ Open. 2025 May 13;15(5):e094973. doi: 10.1136/bmjopen-2024-094973 (PMC12083382; doi:10.1136/bmjopen-2024-094973)
Supplement: online supplemental file 2 [file bmjopen-15-5-s002.docx]

**Supplementary Materials 2**

**Table 1.** Outcomes identified through synthesis with the reasons they are not unique concepts (n=25).

| **Unique Concept** | **Definition** | **Verbatim Outcomes from the Systematic Review** | **Reason not unique** |
| --- | --- | --- | --- |
| Wellbeing | A state of positive feelings and meeting full potential in the world (being the best person you can be in society). It can be measured subjectively and objectively using a salutogenic (positive) approach. ^1^ | Overall wellbeing | Context adjective |
|  |  | General wellbeing | Context adjective |
|  |  | Self-perceived | Methodological adjective (how) |
|  |  | Subjective wellbeing | Methodological adjective (how) |
|  |  | Personal wellbeing | Context adjective |
|  |  | Individual wellbeing | Context adjective |
|  |  | Happiness | Hedonic component |
|  |  | Mental wellbeing | Artificial subgroup |
|  |  | Emotional wellbeing | Artificial subgroup |
|  |  | Psychological wellbeing | Artificial subgroup |
|  |  | Physical wellbeing | Artificial subgroup |
|  |  | Occupational wellbeing | Context adjective |
|  |  | Professional wellbeing | Context adjective |
|  |  | Job related wellbeing | Context adjective |
|  |  | Work related wellbeing | Context adjective |
|  |  | Physician wellbeing | Methodological adjective (who) |
|  |  | Resident wellbeing | Methodological adjective (who) |
|  |  | Organisational wellbeing | Methodological adjective (who) |
|  |  | Unit wellbeing | Methodological adjective (who) |
| Life satisfaction | Separate concept to wellbeing, subjective appraisal of how much the person like the life they lead; one of the indicators of quality of life. ^2 3^ | Satisfied with life | Phrasing |
| Quality of life | Separate concept to wellbeing, subjective appraisal of individuals positioned in life in the context of the culture and value systems in which they live and in relation to their goals, expectations, standards, and concerns. ^4-6^ | Social quality of life | Artificial subgroup |
|  |  | Environmental quality of life | Artificial subgroup |
| Wellness | Separate concept to wellbeing, subjective or objective evaluation of active pursuits of behaviours, choices and lifestyles that lead to a state of holistic health. ^7^ | Health behaviours | Component |
|  |  | Physician wellness | Methodological adjective |
| Optimism | Hopeful transcendence beyond (rising above) immediate circumstances.^8^ | Spiritual wellbeing | Subgroup of wellbeing  Definition of spiritualism the same as optimism, if religion is left out [559] |
| Health | Subjective or objective evaluation of state of complete physical, mental and social wellbeing, not merely the absence of disease of infirmity (the beneficial effects of green spaces for example). ^9 10^ | Mental health | Artificial subgroup |
|  |  | Psychological health | Artificial subgroup |
|  |  | Self-perceived health | Methodological adjective |
|  |  | Satisfaction with health | Methodological adjective |
|  |  | General health status | Phrasing |
| Cognitive function | Objective evaluation of domains such as, but not limited to, Attention, Memory, Processing speed. ^11 12^ | Mental energy | Methodology-self report measure |
| Self-esteem | Self-acceptance, self-worth, sense of coherence (ability to predict events, belief in ability to manage them, that it is worth the effort, ability to be their true self). ^13^ | Confidence | Component |
|  |  | Professional self esteem | Context adjective |
|  |  | Global self esteem | Context adjective |
| Sleep | Subjective or objective evaluation of duration, quality, and sense of feeling rested. ^14^ | Sleep problems | Methodology - pathogenic |
| Financial security | Objective ability to pay for satisfactory accommodation, bills, care of dependents, ability to save for retirement, ability to cope with a sudden fall in income, ability to pay unexpected, but necessary expenses. ^15^ | Household economic wellbeing | Wellbeing subgroup |
| Positive relationships | Subjective or objective assessment of beneficial human connections. ^16^ | Satisfaction with family relations | Methodology |
|  |  | Satisfaction with family support | Methodology |
| Psychological need satisfaction | Subjective or objective assessment of how autonomy, belonging and competence needs have been supported by colleagues (inclusive, positive culture), managers (adequate workforce to allow development), supporting services (IT, administration, legal, occupational health). ^17^ | Job demand support control | Component |
| Job satisfaction | Subjective or objective evaluation of how much they like their choice of work profession, specialism, roles. ^18^ | Occupational choice satisfaction | Methodology |
|  |  | Work satisfaction | Synonym |
|  |  | General job satisfaction | Context adjective |
|  |  | Career satisfaction | Synonym |
|  |  | Professional satisfaction | Synonym |
| Morale | Subjective or objective evaluation of feelings about the future ability of an individual, group or organisation to have and meet shared goals/values. ^19^ | Career morale | Context adjective |
|  |  | Speciality morale | Context adjective |
|  |  | Participation | Methodology |
|  |  | Goal clarity | Component |
| Engagement | Subjective or objective assessment of involvement and absorption with, commitment to, work. ^20^ | Motivational effort | Methodology |
|  |  | Employee job intentions | Methodology |
| Altruism | Subjective or objective evaluation of selfless concern for the wellbeing of others. ^21^ | Altruistic behaviour | Methodology |
| Life work balance | Subjective or objective quantity, quality, and equity of time away from work and at work, the salience/clarity of the roles (the ability to work flexibly). ^22-24^ | Satisfaction with work-life balance | Methodology |
|  |  | Work rest balance | Phrasing |
|  |  | Work pressure | Component |
|  |  | Role behaviour | Methodology |
|  |  | Salience with life roles | Component |
| Professional Development | Subjective or objective assessment of ability to participate and engage with learning and teaching knowledge and skills, and to progress. ^25^ | Skills development | Context adjective |
|  |  | Efficiency | Methodology |
| Identification with work | Subjective or objective assessment of value and meaning assumed by the individual, or a group, at work. ^18^ | Psychological identification with work | Artificial subgroup |
|  |  | Meaning at work | Component |
|  |  | Meaning in work | Component |
| Resilience | Subjective or objective individual, or group level, preservation of, or return to, previous function after exposure to trauma.^26^ | Strain resistant resources | Components |
| Voice and influence | Subjective or objective assessment of ideas, concerns and expectations expressed informing policy and practice.^27^ | Influence | Component |
| Confidence in leadership | Subjective or objective assessment of management competence, transparency and compassion, inclusivity, engagement and empowerment of those they are responsible and accountable for. ^27^ | Supportive leadership | Component |
|  |  | Authority | Synonym |
|  |  | Perceived organisational support | Context component |
| Recognition satisfaction | Subjective or objective evaluation of appreciation by colleagues, patients, public, government (civility, ward rounds/appointments attended and on time). ^28 29^ | Feedback | Methodology |
| Job plan/rota/rotation satisfaction | Subjective or objective evaluation of ability of job plan/rota/rotation to account for the quantity, types, of work (workload), the intensity, duration, of physical, mental and emotional demands and time rest/activities/resources needed to maintain it. ^30^ | Workload | Component |
| Good clinical practice | Subjective or objective assessment of ability to engage with high-risk cases, follow standards where appropriate not rigidly, use diagnostic tests and treatment when clinically indicated and evidence based, not just in case. ^31^ | Self-reported competence | Methodology |

**Table 2**. Unique outcomes for which there were no other terms (n=9)

| **Unique concept** | **Definition** |
| --- | --- |
| Sexual wellbeing | Subjective, or objective, assessment of sense of self and body, appreciating feelings of pleasure and desire, developing and maintaining mutually respectful gender equal relationships, safe and pleasurable sexual interactions.^32^ |
| Personality | Observable enduring characteristics/dispositions/tendencies to engage in certain patterns of behaviour.^8^ |
| Diet | Subjective, or objective, evaluation of the nutritional content, quantity, and timing.^33^ |
| Physical activity | Subjective, or objective, assessment of the ability to participate in physical activity and the quality and quantity of physical exercise. ^34^ |
| Physiological functioning | Objective (snapshot) of body function i.e., Electroencephalography (EEG), Heart Rate Variability, Electro-dermal activity (temperature, sweating), hypothalamic-pituitary axis hormones. ^11 35 36^ |
| Workability | Timely, objective assessment of having occupational competence and virtues, the health required for competence in an appropriate work environment by appropriate occupational health professionals.^37^ |
| Compassion Satisfaction | Subjective evaluation of ability to receive gratification from caregiving to patients, patients’ families, colleagues (satisfaction with non-financial rewards of the work).^38^ |
| Psychological safety | Subjective or objective evaluation of the consequences of taking an interpersonal risk at work (trust, information sharing).^39^ |
| Satisfaction with patient care | Subjective, or objective assessment of quality of health and social care their patients receive. |

**Table 3**. List of unique outcomes identified from other sources (n=9)

| **Unique concept** | **Definition** | **Sources** |
| --- | --- | --- |
| Meaning in life | Separate concept to wellbeing, subjective sense of purpose, engagement with a philosophy of life or life-goals, fulfilment.^40 41^ | WHOQOL SRPB^42^  European Social Survey wellbeing module^43^ |
| Vitality | Relaxed possession of energy (physical, mental and emotional) and vigour, it is not actively strived for.^44^ | European Social Survey wellbeing module^43^  Wilson and Cleary framework (1995)^5 45^  WHO ICF^46^ |
| Novelty | Subjective or objective, growth through new experiences, learning.^47^ | European Social Survey wellbeing module^43^  [from Doctor Engagement Event] |
| Recreational activity | Subjective, or objective, evaluation of the ability to participate and participation in non-work/leisure activities and the qualities of those chosen activities.^48^ | Diener et al (1999) ^45^ |
| Engagement with preventative medicine | Subjective, or objective, assessment of participation in screening programmes they are eligible for and vaccine, timely treatment. | Outcomes for Graduates^49^  NHS People Plan^27^ |
| Financial reward satisfaction | Subjective, or objective, evaluation of ability to receive gratification from financial reward for effort. ^50 51^ | Contract and pension disputes^52 53^ |
| Personal safety | Subjective, or objective, ability to go about work, and get to and from work, free from threat and safe from physical or psychological harm (infection, radiation, bullying, harassment, theft, assault). | NHS People Plan^27^ |
| Self-Care | Subjective, or objective, assessment of behaviours to look after own health and wellbeing at work (taking breaks, time off work for sickness), accessing appropriate support services, adequate resources (estates, workforce, rapid-access self-referral services) to support this. | Outcomes for Graduates^49^ |
| Emotional Intelligence | Subjective, or objective, self-awareness, self-management, social-awareness, and relationship management.^54 55^ | Building and strengthening leadership^56^ |

**Reference:**

1. Simons G, Baldwin DS. A critical review of the definition of 'wellbeing' for doctors and their patients in a post Covid-19 era. *Int J Soc Psychiatry* 2021;67(8):984-91. doi: 10.1177/00207640211032259 [published Online First: 20210709]

2. Ruggeri K, Garcia-Garzon E, Maguire Á, et al. Well-being is more than happiness and life satisfaction: a multidimensional analysis of 21 countries. *Health and Quality of Life Outcomes* 2020;18(1):192. doi: 10.1186/s12955-020-01423-y

3. Veenhoven R. The study of satisfaction with life. In: Saris WE, Veenhoven R, Scherpenzell AC, et al., eds. A comparative study of satisfaction with life in Europe: Eötvös University Press 1996:11-48.

4. British Medical Association. Supporting health and wellbeing at work 2018 [Available from: <https://www.bma.org.uk/media/2076/bma-supporting-health-and-wellbeing-at-work-oct-2018.pdf>.

5. Wilson IB, Cleary PD. Linking Clinical Variables With Health-Related Quality of Life: A Conceptual Model of Patient Outcomes. *JAMA* 1995;273(1):59-65. doi: 10.1001/jama.1995.03520250075037

6. Ferrans CE, Zerwic JJ, Wilbur JE, et al. Conceptual Model of Health-Related Quality of Life. *Journal of Nursing Scholarship* 2005;37(4):336-42. doi: <https://doi.org/10.1111/j.1547-5069.2005.00058.x>

7. Global Wellness Institute. What is wellness? n.d. [Available from: <https://globalwellnessinstitute.org/what-is-wellness/20> September 2020.

8. Sharpe JP, Martin NR, Roth KA. Optimism and the Big Five factors of personality: Beyond Neuroticism and Extraversion. *Personality and Individual Differences* 2011;51(8):946-51. doi: 10.1016/j.paid.2011.07.033

9. World Health Organization (WHO). Preamble to the Constitution of the World Health Organization as adopted by the International Health Conference. New York, 19-22 June, 1946; Signed on 22 July 1946 by the representatives of 61 States (Official Records of the World Health Organization, no. 1, p.100) and entered into force on 7 April 1948. Geneva, Switzerland, 1948.

10. Moore THM, Kesten JM, Lopez-Lopez JA, et al. The effects of changes to the built environment on the mental health and well-being of adults: Systematic review. *Health Place* 2018;53:237-57. doi: 10.1016/j.healthplace.2018.07.012 [published Online First: 20180906]

11. Yetton BD, Revord J, Margolis S, et al. Cognitive and Physiological Measures in Well-Being Science: Limitations and Lessons. *Frontiers in Psychology* 2019;10 doi: 10.3389/fpsyg.2019.01630

12. Lieberman HR. Cognitive methods for assessing mental energy. *Nutritional Neuroscience* 2007;10(5-6):229-42. doi: 10.1080/10284150701722273

13. Stets JE, Burke PJ. Self-Esteem and Identities. *Sociological Perspectives* 2014;57(4):409-33. doi: 10.1177/0731121414536141

14. Harvey AG, Stinson K, Whitaker KL, et al. The Subjective Meaning of Sleep Quality: A Comparison of Individuals with and without Insomnia. *Sleep* 2008;31(3):383-93. doi: 10.1093/sleep/31.3.383

15. Office for National Statistics. Personal and economic well-being in Great Britain: May 2021 2021 [Available from: <https://www.ons.gov.uk/peoplepopulationandcommunity/wellbeing/bulletins/personalandeconomicwellbeingintheuk/may202119> December 2021.

16. Viljoen J. Investigating the use of the PERMA theory of wellbeing in the New Zealand General Social Survey. Massey University, 2018.

17. Vansteenkiste M, Ryan RM, Soenens B. Basic psychological need theory: Advancements, critical themes, and future directions. *Motivation and Emotion* 2020;44(1):1-31. doi: 10.1007/s11031-019-09818-1

18. Miscenko D, Day DV. Identity and identification at work. *Organizational Psychology Review* 2016;6(3):215-47. doi: 10.1177/2041386615584009

19. Hardy B. Morale: definitions, dimensions and measurements. University of Cambridge, 2010.

20. Kuok ACH, Taormina RJ. Work engagement: Evolution of the concept and a new inventory. *Psychological Thought* 2017;10(2):262-87. doi: <https://doi.org/10.5964/psyct.v10i2.236>

21. Gleichgerrcht E, Decety J. Empathy in clinical practice: how individual dispositions, gender, and experience moderate empathic concern, burnout, and emotional distress in physicians. *PLoS One* 2013;8(4):e61526. doi: 10.1371/journal.pone.0061526 [published Online First: 20130419]

22. Brough P, Timms C, Chan XW, et al. Work-Life Balance: Definitions, Causes and Consequences. In: Theorell T, ed. Handbook of Socioeconomic Determinants of Occupational Health. Switzerland: Springer Nature, 2020.

23. Nitzsche A, Neumann M, Groß SE, et al. Recovery opportunities, work–home conflict, and emotional exhaustion among hematologists and oncologists in private practice. *Psychology, Health & Medicine* 2017;22(4):462-73. doi: 10.1080/13548506.2016.1237666

24. Clark SC. Work Cultures and Work/Family Balance. *Journal of Vocational Behavior* 2001;58(3):348-65. doi: 10.1006/jvbe.2000.1759

25. Oliveira-Silva L, Porto J, Arnold J. Professional fulfilment: Concept and instrument proposition. *Psico-USF* 2019;27:27-39.

26. McGeary DD. Making sense of resilience. *Mil Med* 2011;176(6):603-4. doi: 10.7205/milmed-d-10-00480

27. NHS England. WE ARE THE NHS: People Plan for 2020/2021 - action for us all, 2020.

28. Rippon S, Hopkins T. Head, hands and heart: asset-based approaches in health care: The Health Foundation, 2015.

29. Proctor C, Tweed R, Morris D. The Rogerian Fully Functioning Person:A Positive Psychology Perspective. *Journal of Humanistic Psychology* 2016;56(5):503-29. doi: 10.1177/0022167815605936

30. General Medical Council. Training environments 2018: Key findings from the national training surveys, 2018.

31. Ortashi O, Virdee J, Hassan R, et al. The practice of defensive medicine among hospital doctors in the United Kingdom. *BMC Medical Ethics* 2013;14(1):42. doi: 10.1186/1472-6939-14-42

32. Kågesten A, van Reeuwijk M. Adolescent Sexual Wellbeing: A Conceptual Framework, 2021.

33. Cvejic E, Parker G, Harvey SB, et al. The health and well-being of Australia's future medical doctors: protocol for a 5-year observational cohort study of medical trainees. *BMJ Open* 2017;7(9):e016837. doi: 10.1136/bmjopen-2017-016837 [published Online First: 20170911]

34. National Institute for Health and Care Excellence. Physical activity in the workplace: Public health guideline [PH13], 2008.

35. Mattsson S, Fast-Berglund Å, Åkerman M. Assessing Operator Wellbeing through Physiological Measurements in Real-Time—Towards Industrial Application. *Technologies* 2017;5(4):61.

36. Tsigos C, Kyrou I, Kassi E, et al. Stress: Endocrine Physiology and Pathophysiology. In: Feingold K, Anawalt B, Blackman M, eds. Endotext. South Dartmouth (MA): MDText.com, Inc., 2020.

37. Tengland P-A. The Concept of Work Ability. *Journal of Occupational Rehabilitation* 2011;21(2):275-85. doi: 10.1007/s10926-010-9269-x

38. Hooper C, Craig J, Janvrin DR, et al. Compassion satisfaction, burnout, and compassion fatigue among emergency nurses compared with nurses in other selected inpatient specialties. *J Emerg Nurs* 2010;36(5):420-7. doi: 10.1016/j.jen.2009.11.027 [published Online First: 20100518]

39. Edmondson AC, Lei Z. Psychological Safety: The History, Renaissance, and Future of an Interpersonal Construct. *Annual Review of Organizational Psychology and Organizational Behavior* 2014;1(1):23-43. doi: 10.1146/annurev-orgpsych-031413-091305

40. Hill CE, Kline KV, Miller M, et al. Development of the Meaning in Life Measure. *Counselling Psychology Quarterly* 2019;32(2):205-26. doi: 10.1080/09515070.2018.1434483

41. Morgan J, Farsides T. Measuring Meaning in Life. *Journal of Happiness Studies* 2009;10(2):197-214. doi: 10.1007/s10902-007-9075-0

42. Skevington SM, Gunson KS, O'Connell KA. Introducing the WHOQOL-SRPB BREF: developing a short-form instrument for assessing spiritual, religious and personal beliefs within quality of life. *Qual Life Res* 2013;22(5):1073-83. doi: 10.1007/s11136-012-0237-0 [published Online First: 20120727]

43. Huppert FA, So TT. Flourishing Across Europe: Application of a New Conceptual Framework for Defining Well-Being. *Soc Indic Res* 2013;110(3):837-61. doi: 10.1007/s11205-011-9966-7 [published Online First: 20111215]

44. Guérin E. Disentangling Vitality, Well-Being, and Quality of Life: A Conceptual Examination Emphasizing Their Similarities and Differences With Special Application in the Physical Activity Domain. *Journal of Physical Activity and Health* 2012;9(6):896-908. doi: 10.1123/jpah.9.6.896

45. Diener E, Suh EM, Lucas RE, et al. Subjective well-being: Three decades of progress. *Psychological Bulletin* 1999;125(2):276-302. doi: 10.1037/0033-2909.125.2.276

46. World Health Organization. Towards a Common Language for Functioning, Disability and Health ICF. Geneva, 2002.

47. González-Cutre D, Sicilia Á, Sierra AC, et al. Understanding the need for novelty from the perspective of self-determination theory. *Personality and Individual Differences* 2016;102:159-69. doi: 10.1016/j.paid.2016.06.036

48. Cydulka RK, Korte R. Career satisfaction in emergency medicine: the ABEM Longitudinal Study of Emergency Physicians. *Ann Emerg Med* 2008;51(6):714-22 e1. doi: 10.1016/j.annemergmed.2008.01.005 [published Online First: 20080408]

49. General Medical Council. Outcomes for graudates 2018, 2018.

50. Herzer KR, Pronovost PJ. Physician Motivation: Listening to What Pay-for-Performance Programs and Quality Improvement Collaboratives Are Telling Us. *Jt Comm J Qual Patient Saf* 2015;41(11):522-8. doi: 10.1016/s1553-7250(15)41069-4

51. Bimpong KAA, Khan A, Slight R, et al. Relationship between labour force satisfaction, wages and retention within the UK National Health Service: a systematic review of the literature. *BMJ Open* 2020;10(7):e034919. doi: 10.1136/bmjopen-2019-034919 [published Online First: 20200721]

52. Rimmer A. Junior doctor strikes had a “significant impact” on services but did not increase deaths, study finds. *Bmj* 2018 doi: 10.1136/bmj.k782

53. Mahase E. Pensions: BMA challenges government's decision to make doctors pay for illegal reforms. *BMJ* 2021;375:n2809. doi: 10.1136/bmj.n2809 [published Online First: 20211116]

54. Cherniss C. Emotional Intelligence: Toward Clarification of a Concept. *Industrial and Organizational Psychology* 2010;3(2):110-26. doi: 10.1111/j.1754-9434.2010.01231.x [published Online First: 2015/01/07]

55. Mintz LJ, Stoller JK. A systematic review of physician leadership and emotional intelligence. *J Grad Med Educ* 2014;6(1):21-31. doi: 10.4300/JGME-D-13-00012.1

56. NHS England. Building and Strengthening Leadership: Leading with Compassion, 2014.
