# Supplementary material for: Which outcomes should be included in a core outcome set for capturing and measuring doctor well-being? A Delphi study
Source: BMJ Open. 2025 May 13;15(5):e094973. doi: 10.1136/bmjopen-2024-094973 (PMC12083382; doi:10.1136/bmjopen-2024-094973)
Supplement: online supplemental file 3 [file bmjopen-15-5-s003.docx]

**Supplementary Materials 3**

Stakeholder organisations

- The symbol > is used for n as the classification of the individuals and organisations into stakeholder groups was undertaken by the author.

1. Those who could use COS-DR in their research - based on that, they have previously published doctor well-being research individually (n>15) or as an organisation (n>5).

- British Medical Association
- Health Education and Improvement Wales
- Health Education England
- Kings’ Fund
- Practitioner Health Programme

1. Organisations that measure doctor well-being in the NHS in the UK every year (n>3).

- British Medical Association
- General Medical Council
- Practitioner Health Programme
- Royal College of Physicians

1. Professionals with experience of managing doctor wellbeing individually, including doctors, nurses, psychologists, and liaison advisors (n>8) and organisations (n>2).

- British Medical Association
- Practitioner Health Programme

1. Doctors individually (n=48) and organisations representing them (n>12)

- Association of Anaesthetists
- British Association of Physicians of Indian Origin
- British Medical Association
- Faculty of Occupational Medicine
- Health Education and Improvement Wales
- Health Education England
- Kings’ Fund
- Practitioner Health Programme
- Royal College of General Practitioners
- Royal College of Paediatric and Child Health
- Royal College of Physicians.
