# Supplementary material for: Which outcomes should be included in a core outcome set for capturing and measuring doctor well-being? A Delphi study
Source: BMJ Open. 2025 May 13;15(5):e094973. doi: 10.1136/bmjopen-2024-094973 (PMC12083382; doi:10.1136/bmjopen-2024-094973)
Supplement: Supplementary file 6 [file bmjopen-2024-094973.reviewer_comments.pdf]

PEER REVIEW HISTORY

BMJ Open publishes all reviews undertaken for accepted manuscripts. Reviewers are asked to complete a checklist review form and are provided with free text boxes to elaborate on their assessment. These free text comments are reproduced below.

ARTICLE DETAILS

Title (Provisional)

Which outcomes should be included in a core outcome set for capturing and measuring doctor wellbeing? A Delphi study

Authors

Simons, Gemma; Klepacz, Naomi; Baldwin, David

VERSION 1 - REVIEW

**Reviewer**	**1**
**Name**	**Hayes, Blánaid**
**Affiliation**	**Beaumont Hospital**
**Date**	**28-Jan-2025**
**COI**	**None**

I commend the authors on this paper. The topic is timely, indeed one might say long overdue! It is nicely laid out and very well written. Any comments I've made are very minor and I recommend it for publication.

Minor comments for consideration:

1. Line 317: 7% is a small enough representation of junior doctors, though I appreciate that on the ground, it can be difficult to engage them in research such as this, given their clinical commitments. It's possible that their ranking of core outcomes would be different to their more experienced senior colleagues and might have influenced the final consensus of 7 core outcomes

2.I agree with the authors that the gamut of instruments currently available to measure a wide range of positive and negative variables relating to wellbeing and mental health can be bewildering and makes it challenging to choose options at the research stage as well as to compare results between published studies. This paper will guide researchers to a set of seven core outcomes and I note the researchers observation in line 345 regarding further research. Meanwhile, it would be helpful if it were easy to deduce from the references cited what some of these instruments were e.g. those used in the NHS staff survey pertaining to life work balance, wellbeing, health and morale.

-the issue of life-work balance is clear with 2 explicit questions in #6

-likewise the issue of morale, which utilises the questions relating to intention to leave in #26

-Which items related to health and wellbeing were less clear, despite headings including the terms (#11 and #12). Question 11 asks about the organisation's action and explores msk problems, stress and inability to work due to being unwell. Question 12 includes the 2 items (a&b) often used as a 'mini-burnout' questionnaire.

I don't see a question or set of questions exploring wellbeing per se (e.g. WHO-5). One could say the same about health.

I think it would be helpful to future researchers in the field if the authors could clarify which of the NHS survey questions they viewed as measuring health and wellbeing respectively.

**Reviewer**	**2**
**Name**	**Creese, Jennifer**
**Affiliation**	**University of Leicester, Department of Health Sciences**
**Date**	**20-Feb-2025**
**COI**	**None**

Thank you for the opportunity to review this paper, which details the rigorous development of a core outcome set (COS) for measuring doctor wellbeing. The paper provides a comprehensive overview of the pressures on wellbeing for doctors in a UK/NHS context, and uses a robust methodology to arrive through consensus at a co-created standard set of outcomes. This paper, and the COS it lays out, will be an important asset to research and research-informed practice around employee wellbeing, specifically to doctors but with broader implications. It has definitive potential to inform a further development of validated outcome measures to accompany. The scholarly write-up of this exercise and tool is of very high quality. I have no hesitation in recommending its acceptance for publication, though I do make some minor comments that might be easily revised:

1) There might be broader utility for this work if the authors could clarify, in the introduction, that doctor poor wellbeing is a global phenomenon. The references they draw on do come from a variety of settings (mainly European) but the text straight away positions the idea of doctor wellbeing within the NHS staff survey, which immediately limits the view/discussion to a UK only context. It could be a simple fix to say “Doctors globally” or “Doctors in health systems across the world” in the first sentence, then “In a UK context” when narrowing scope to NHS staff survey findings. Similarly when talking about impacts and costs.

2) The authors state that “A salutogenic approach…should be preferred when considering doctor wellbeing” – expand a little more on why specifically

3) Only a minor thing – Table 2 refers to “life work balance” – work life balance is by and large the preferred terminology in most literature, and though the rendering “life work balance” is beginning to take on some prominence, it does have a deeper critical philosophical argument to it in terms of the nature and primacy of work (i.e. which gets put first is the main one) which is a much deeper debate than I expect the authors wish to get into at this stage. Perhaps this was discussed in the authors’ systematic review; if so could a footnote be used to indicate that the terminology is contested but has

been established in this way for a reason elsewhere, and include the reference? If this was just a surface level decision, with no attempt to get into the deeper terminological debate, perhaps the authors might make life easier on themselves by changing to WLB (this would also increase discoverability/search optimisation)

VERSION 1 - AUTHOR RESPONSE

Reviewer Comments	Response
**Reviewer 1**	
I commend the authors on this paper. The topic is timely, indeed one might say long overdue! It is nicely laid out and very well written. Any comments I've made are very minor and I recommend it for publication.	We appreciate the reviewer's positive feedback and thoughtful assessment of our work. We further appreciate recognition of the paper's relevance and clarity, and we have carefully addressed the minor comments in the following sections.
1. Line 317: 7% is small enough representation of junior doctors, though I appreciate that on the ground, it can be difficult to engage them in research such as this, given their clinical commitments. It's possible that their ranking of core outcomes would be different to their more experienced senior colleagues and might have influenced the final consensus of 7 core outcomes.	Thank you for raising this point. We have added the following text to our discussion. [Lines 411-413] 'However, we acknowledge that some groups – for example resident doctors – were underrepresented and that stakeholders outside the present panel might have differing views from those of the final consensus.'
2. I agree with the authors that the gamut of instruments currently available to measure a wide range of positive and negative variables relating to wellbeing and mental health can be bewildering and makes it challenging to choose options at the research stage as well as to compare results between published studies. This paper will guide researchers to a set of seven core outcomes and I note the researchers observation in line 345 regarding further research. Meanwhile, it would be helpful if it were easy to deduce from the references cited what some of these instruments were e.g. those used in the NHS staff survey pertaining to life work balance, wellbeing, health and morale.<br>- The issue of life-work balance is clear with 2 explicit questions in #6<br>- Likewise the issue of morale, which utilises the questions relating to intention to leave in #26<br>- Which items related to health and wellbeing were less clear, despite headings including the terms (#11 and #12). Question 11 asks about the organisation's action and explores msk problems, stress and inability to work due to being unwell. Question 12 includes the 2 items (aCb) often used as a 'mini-burnout' questionnaire.<br>I don't see a question or set of questions exploring	Thank you for your thoughtful feedback. We agree that it is important for readers to clearly understand how the NHS Staff Survey questions relate to the COS. To address this, we have adjusted line [Lines 366-379] to provide greater clarity on the alignment between COS outcomes and specific survey questions.<br><br>The choice of outcome measures instruments for the core outcome set is the focus of our ongoing research and we hope to address the points your raise more thoroughly in that work. We are that we glad we are not the only ones to notice the parallels between the core outcome set and the NHS staff survey questions, but also the lack of a clear wellbeing question. We have been in communication with the NHS staff survey team regarding question choice and measurement instruments but obtaining detailed insights have proven challenging. Therefore, the discussion presented reflected the authors interpretation of how existing NHS staff survey questions align with the COS with a more detailed discussion to follow in our ongoing work.

wellbeing per se (e.g. WHO-5). Once could say the same about health.<br>I think it would be helpful to future researchers in the field if the authors could clarify which of the NHS survey questions they viewed as measuring health and wellbeing respectively.	

**Reviewer 2**	
Thank you for the opportunity to review this paper, which details the rigorous development of a core outcome set (COS) for measuring doctors wellbeing. The paper provides a comprehensive overview of the pressures on wellbeing for doctors in a UK/NHS context, and uses a robust methodology to arrive through consensus at a co-created standard set of outcomes. This paper, and the COS it lays out, will be an important asset to research and research-informed practice around employee wellbeing, specifically to doctors but with broader implications. It has definitive potential to inform a further development of validated outcome measures to accompany. The scholarly write-up of this exercise and tool is of very high quality. I have no hesitation in recommending its acceptance for publication, though I do make some minor comments that might be easily revised:	We appreciate the reviewer's thoughtful and detailed feedback. We are pleased that the rigour of our methodology and the relevance of our work within the UK/NHS context have been recognised and appreciate the reviewer's assessment on the potential impact of the core outcome set and the quality of our write-up. We have carefully addressed the minor comments in the following sections and thank the reviewer for their valuable input.
1. There might be broader utility for this work if the authors could clarify, in the introduction, that doctor poor wellbeing is a global phenomenon. The references they draw on do come from a variety of settings (mainly European) but the text straight away positions the idea of doctor wellbeing within the NHS staff survey, which immediately limits the view/discussion to a UK only context. It could be a simple fix to say "Doctors globally" or "Doctors in health systems across the world" in the first sentence, then "In a UK context" when narrowing scope to NHS staff survey findings. Similarly when talking about impact and costs.	Thank you for this feedback. We have done as Dr Creese suggests and have added the word globally to our first sentence [Line 120] of the introduction and to our third sentence we have added 'In the UK context' [Line 123].
2. The authors state that "A salutogenic approach…should be preferred when considering doctor wellbeing" – expand a little more on why specifically	We have added the following: "A salutogenic approach is a positive approach that looks prospectively at how to create, enhance, and improve wellbeing; as opposed to a pathogenic approach that looks retrospectively at the disease burden of doctors (usually mental health diagnoses)." [Lines 166-168]

3. Only a minor thing – Table 2 refers to "life work balance" – work life balance is by and large the preferred terminology in most literature, and though the rendering "life work balance" is beginning to take on some prominence, it does have a deeper critical philosophical argument to it in terms of the nature and primacy of work (i.e. which gets put first is the main one) which is a much deeper debate that I expect the authors wish to get into at this stage. Perhaps this was discussed in the authors' systematic review; if so could a footnote be used to indicate that the terminology is contested but has been established in this way for a reason elsewhere, and include the reference? If this was just a surface level decision, with no attempt to get into the deeper terminological debate, perhaps the authors might make life easier on themselves by changing to WLB (this would also increase discoverability/search optimisation)	Thank you for picking up this important nuance. It was a conscious decision to value life before work. We will add a footnote to Table 1 saying 'work life balance is the preferred terminology in most literature. Placing life before work was a conscious decision by the authors for this outcome and will impact the future outcome measurement instrument selection. Further discussion can be found it Simons (2022).'
